# Myopericarditis After Combined Idarubicin and Quizartinib in FLT3-ITD–Positive AML

**DOI:** 10.1016/j.jaccas.2026.108334

**Published:** 2026-05-14

**Authors:** Zachary R. Spahr, Moises A. Vasquez, Adarshvardhan Tangella, Mohammad Saad Husain, Allen J. Taylor

**Affiliations:** a Department of Medicine, MedStar Georgetown University Hospital/Georgetown University School of Medicine, Washington, DC, USA; b MedStar Heart and Vascular Institute, Washington, DC, USA; c MedStar Washington Hospital Center/Georgetown University Hospital, Washington, DC, USA

**Keywords:** cardiomyopathy, electrocardiogram, imaging

## Abstract

**Background:**

Anthracyclines are key components of induction therapy for acute myelogenous leukemia with well-understood cardiotoxicity. Quizartinib inhibits FLT3 and has been shown to potentiate apoptosis and promote maladaptive remodeling after myocardial infarction in mice; however, the impact of FLT3 inhibition on a heart already exposed to anthracyclines is poorly understood.

**Case Summary:**

A 64-year-old man with acute myelogenous leukemia developed fever, hypotension, troponin I elevation, and diffuse ST-segment elevation after treatment with cytarabine and idarubicin followed by quizartinib. Cardiac magnetic resonance imaging showed late gadolinium enhancement consistent with myopericarditis and a new reduced left ventricular ejection fraction. Quizartinib was stopped, and the patient received colchicine and guideline-directed medical therapy. Quizartinib was successfully reintroduced at a lower dose without recurrence of cardiac injury.

**Discussion:**

This case illustrates a “two-hit” mechanism resulting in acute myopericarditis. The reversibility of the injury and the successful rechallenge emphasize the potential role of dose modification and close monitoring when combining targeted therapies with anthracyclines.

**Take-Home Messages:**

Drug-induced myopericarditis should be suspected when cardiotoxic agents are administered sequentially. Rechallenge with a reduced dose may be feasible.

## Visual Summary

**Figure:**
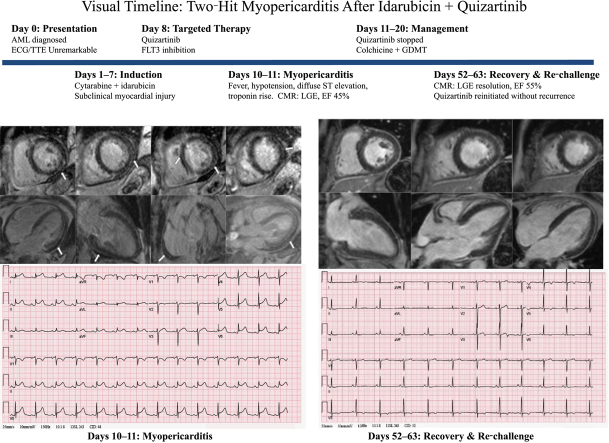

**Visual Summary:**
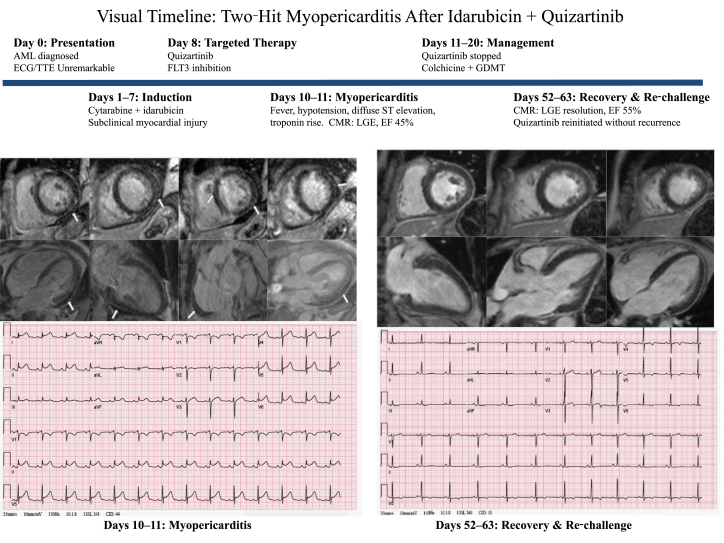
Myopericarditis After Sequential Idarubicin and Quizartinib White arrows indicate area of myocardial late gadolinium enchantment. AML = acute myelogenous leukemia; CMR = cardiac magnetic resonance; ECG = electrocardiogram; EF = ejection fraction; FLT3 = Fms-like tyrosine kinase 3; GDMT = guideline-directed medical therapy; LGE = late gadolinium enhancement; TTE = transthoracic echocardiogram.

## History of Presentation

A 64-year-old man presented to the emergency department with 3 weeks of progressive fatigue and generalized weakness. Vital signs on arrival were stable, and physical examination was unremarkable. A complete blood count showed a white blood cell count of 91 × 10^9^/L. A bone marrow biopsy confirmed acute myelogenous leukemia (AML) with an Fms-like tyrosine kinase 3 (FLT3)–internal tandem duplication (FLT3-ITD) mutation. The patient was commenced on standard “7 + 3” induction therapy with cytarabine and idarubicin. Baseline investigations included a 12-lead electrocardiogram (ECG), which showed normal sinus rhythm without conduction abnormalities or ST-segment changes, and a transthoracic echocardiogram (TTE) demonstrating a structurally normal left ventricle with an ejection fraction of 60% to 65% and mild mitral and tricuspid regurgitation. On day 8 of induction, he received the first dose of quizartinib (200 mg daily) as FLT3-targeted therapy.

**Take-Home Messages**

• When multiple cardiotoxic agents are administered sequentially, clinicians should consider drug-induced myopericarditis and obtain timely imaging and biomarker assessment.
• Fms-like tyrosine kinase 3 (FLT3) inhibitors may precipitate acute myopericarditis by blocking cardioprotective FLT3 signaling in a heart primed by anthracycline-induced oxidative and genotoxic stress.
• Reintroduction of FLT3 inhibitors at lower doses may be feasible after complete resolution of inflammatory injury, but requires close cardiac monitoring and multidisciplinary decision making.

**Figure 1:**
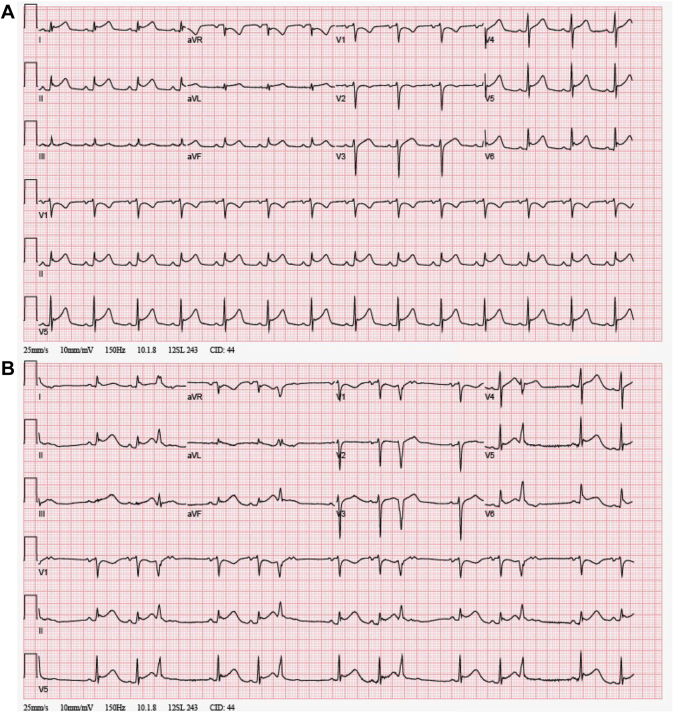
12-Lead Electrocardiogram (A) Initial 12-lead electrocardiogram at day 10 showing diffuse concave ST-segment elevations in the precordial and inferior leads. (B) Follow-up electrocardiogram at day 12 showing persistent ST-segment elevation with ventricular trigeminy.

Two days later (treatment day 10), the patient developed fever (38.7 °C), hypotension, and malaise. He denied chest pain. The repeat ECG revealed new diffuse ST-segment elevation most pronounced in the precordial and inferior leads (Figure 1), with the subsequent ECG showing ventricular trigeminy. Vital signs showed a blood pressure of 86/54 mm Hg and a heart rate of 96 beats/min. Physical examination remained otherwise unremarkable, with no pericardial rub or muffled heart sounds.

## Past Medical History

The patient's past medical history included long-standing hypertension controlled with amlodipine 5 mg daily. He had no known coronary artery disease and no history of cardiac symptoms. There was no family history of premature cardiovascular disease. He did not smoke or consume alcohol. The baseline ECG and TTE before the initiation of chemotherapy were normal.

## Differential Diagnosis

In the context of an immunocompromised patient receiving multiagent chemotherapy, the differential diagnosis for acute troponin elevation and diffuse ST-segment elevation included viral myocarditis, septic cardiomyopathy, acute pericarditis, stress cardiomyopathy, acute coronary syndrome, and drug-induced cardiac injury. The absence of chest pain, lack of regional wall motion abnormalities on echocardiography, and nonischemic pattern of late gadolinium enhancement on cardiac magnetic resonance imaging (CMR) argued against acute coronary syndrome. Septic shock and sepsis-associated cardiomyopathy were considered; however, serial blood cultures remained negative and there was no infectious source. The temporal association with the introduction of quizartinib, as well as prior exposure to idarubicin 7 days earlier, suggested drug-induced myopericarditis due to sequential cardiotoxic hits. Acute anthracycline toxicity was considered, but was argued against by the temporal association of quizartinib initiation, subepicardial and mid-myocardial late gadolinium enhancement, and rapid recovery suggested acute inflammation rather than progressive damage.

## Investigations

**Figure 2:**
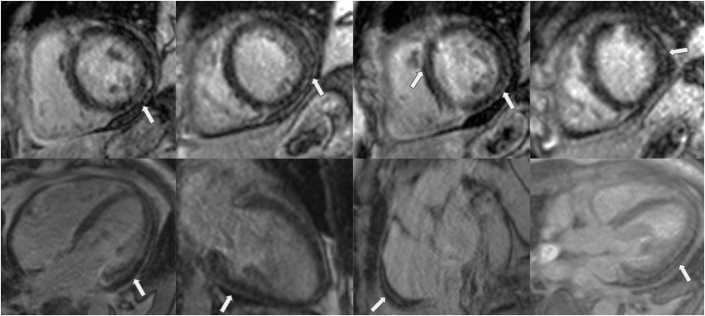
Cardiac Magnetic Resonance Imaging on Day 11 Cardiac magnetic resonance imaging obtained on day 11 demonstrating mildly dilated left ventricle and patchy subepicardial and mid-myocardial late gadolinium enhancement in 4 segments, consistent with acute myopericarditis. T2-weighted imaging showed increased myocardial signal intensity. White arrows indicate area of myocardial late gadolinium enchantment.

Initial laboratory tests revealed a high-sensitivity troponin I concentration of 260 ng/L (reference range: <34 ng/L), which subsequently peaked at 420 ng/L. C-reactive protein was elevated at 120 mg/L. The ECG showed diffuse concave ST-segment elevation without reciprocal depression. A TTE obtained on day 10 demonstrated an ejection fraction of 55% with subtle apical hypokinesis; there was no significant pericardial effusion. Cardiac magnetic resonance performed the following day revealed an ejection fraction of 45% with a mildly dilated left ventricle and patchy subepicardial and mid-myocardial late gadolinium enhancement in 4 segments, consistent with acute myopericarditis (Figure 2). T2-weighted imaging showed increased myocardial signal intensity satisfying the modified Lake Louise criteria. There was no evidence of ischemia or infarction. The results of viral polymerase chain reaction testing (enterovirus, adenovirus, parvovirus B19, Epstein-Barr virus, and cytomegalovirus) and serial blood cultures were negative. The diffuse concave ST-segment pattern without reciprocal changes, absent regional wall motion abnormalities, and subepicardial CMR enhancement made coronary disease unlikely, and coronary angiography was deferred.

## Management

Upon diagnosis of myopericarditis, quizartinib was immediately discontinued. The patient was started on colchicine 0.6 mg twice daily to treat the pericardial inflammation. Guideline-directed medical therapy for heart failure with mildly reduced ejection fraction was initiated, including sacubitril/valsartan 24/26 mg twice daily and metoprolol succinate 25 mg daily. Idarubicin and cytarabine were not reintroduced, given the conclusion of induction. Empirical broad-spectrum antibiotics were continued until cultures remained negative. Serial troponin levels declined and ST-segment abnormalities resolved over the following week. The patient remained hemodynamically stable and asymptomatic. The repeat TTE on day 20 showed improvement in global systolic function and no pericardial effusion.

## Outcome and Follow-Up

**Figure 3:**
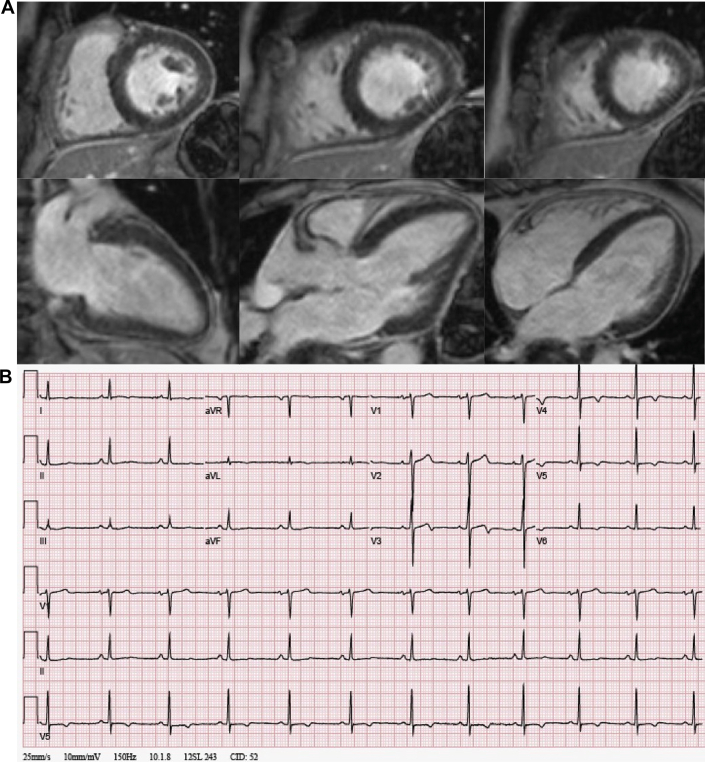
Cardiac Magnetic Resonance Imaging on Day 63 (A) Cardiac magnetic resonance imaging obtained on day 63 demonstrating resolution of late gadolinium enhancement. (B) Follow-up electrocardiogram on day 61 demonstrating resolution of ST-segment elevations.

By day 52, repeat cardiac magnetic resonance demonstrated recovery of left ventricular ejection fraction to 55% and near-complete resolution of increased T2 signal and late gadolinium enhancement, except for a small residual focus (Figure 3). The patient was readmitted 1 month later for consolidation therapy with high-dose cytarabine. Given the lack of alternative targeted options for FLT3-ITD AML and after detailed multidisciplinary discussion, quizartinib was reintroduced at a reduced dose of 60 mg daily with close cardiac monitoring. There was no recurrence of fever, troponin elevation, or ECG changes, and the patient completed consolidation without further cardiac events. At 3-month follow-up, he remained asymptomatic, with stable left ventricular function on echocardiography.

## Discussion

### Mechanistic “two-hit” hypothesis

Anthracyclines are cornerstone agents in AML therapy but are notorious for cardiotoxicity. They bind topoisomerase II enzymes to cause DNA double-strand breaks and generate reactive oxygen species through redox cycling.^1^ These processes contribute to acute and chronic cardiomyopathy and mitochondrial dysfunction.^1^ Experimental models demonstrate that FLT3 is expressed in cardiomyocytes and that activating the receptor with its ligand reduces infarct size, ameliorates remodeling, and protects against oxidative stress–induced apoptosis via protein kinase B signaling.^2^ Consequently, FLT3 inhibitors may remove this endogenous protective pathway. A recent preclinical study found that quizartinib exposure promoted apoptosis and maladaptive remodeling after myocardial infarction, and in vitro it potentiated hydrogen peroxide–induced cell death via a p38-dependent mechanism.^3^^,^^4^ Although chronic dose-dependent cardiotoxicity is the most recognized complication of anthracycline therapy, acute myocarditis is a rare but described manifestation and may contribute independently or synergistically to cardiac injury.

In our patient, the first hit likely occurred during idarubicin exposure, which produced subclinical myocardial injury through DNA damage and reactive oxygen species generation. The second hit occurred when quizartinib inhibited the FLT3 receptor, disabling protein kinase B-dependent prosurvival signaling and allowing oxidative and genotoxic stress to trigger clinically apparent myopericarditis. The rapid improvement after stopping quizartinib and initiating anti-inflammatory and heart failure therapy supports this mechanism. Quizartinib has a half-life of ∼81 hours; steady-state is typically achieved after 15 days. Symptom onset on day 10 (after only 2 doses) suggests subtherapeutic levels triggered myocarditis. Successful rechallenge at 60 mg (70% reduction) supports dose-dependent toxicity rather than hypersensitivity. It also underscores the importance of cautious reintroduction under careful monitoring when alternative targeted agents are lacking.

### Comparison with published literature

Clinical trials of quizartinib, such as QuANTUM-First, have primarily identified QT prolongation as the predominant cardiac toxicity, with torsades de pointes and cardiac arrest occurring in <1% of patients.^5^ Reports of structural cardiac injury are extremely rare. To date, there are no published case reports of quizartinib-associated myopericarditis.^6^ However, case reports of myocarditis with first-generation FLT3 inhibitors exist. Di Lillo et al^7^ described a case of a 24-year-old patient who developed focal myocarditis 3 weeks after receiving gilteritinib; his cardiac function improved after drug cessation and steroid therapy. Zakhour et al^8^ reported 2 cases of delayed systolic dysfunction developing several months after the initiation of gilteritinib. Recently, Kalkan et al^9^ described severe perimyocarditis within 5 days of starting midostaurin in a young adult with FLT3-ITD AML. All 3 cases highlight that targeted FLT3 inhibition can precipitate inflammatory cardiac injury, but none involved quizartinib, nor the immediate onset seen in our patient. Thus, our case broadens awareness of class-wide vulnerability and underscores the need for vigilance.

### Clinical implications

Clinicians should recognize that the safety profile of targeted therapies cannot be assessed in isolation. When used sequentially with cytotoxic agents, targeted inhibitors may precipitate synergistic cardiotoxicity. The overlap phase between therapies represents a particularly vulnerable period for cardiotoxicity and should prompt heightened clinical surveillance. In practice, baseline cardiac evaluation, including ECG and echocardiography, should be performed before and during therapy. Biomarkers such as troponin and natriuretic peptides can detect subclinical injury. Acute myopericarditis can present without typical chest pain; constitutional symptoms may dominate when inflammation is severe. If symptoms, ECG changes, or biomarker elevations occur, early use of CMR may clarify diagnosis and avoid unnecessary coronary angiography. Management involves prompt discontinuation of the offending agent, anti-inflammatory therapy, and initiation of guideline-directed heart failure therapy. Re-exposure can be considered when benefits outweigh risks, but only after complete recovery and with dose reduction and careful monitoring. Further studies are needed to better define the optimal timing and dosing of quizartinib rechallenge after episodes of myopericarditis to guide structured clinical management.

## Conclusions

This case illustrates a potential mechanism of myopericarditis in the setting of sequential exposure to anthracyclines and FLT3 inhibition. Although other FLT3 inhibitors have been associated with myocarditis in isolated reports, our case highlights the potential for synergistic toxicity with sequential anthracycline exposure. The pathophysiology is consistent with a “two-hit” model in which anthracycline-induced oxidative and genotoxic stress primes the myocardium and subsequent FLT3 inhibition removes cardioprotective signaling, leading to inflammatory injury. Awareness of this synergistic toxicity can prompt early recognition, appropriate management, and informed decision making about rechallenging with targeted therapy. Further case reports and mechanistic studies are needed to determine whether myopericarditis observed in this case represents a quizartinib-specific effect, a class effect of FLT3 inhibitors, or a unique toxicity of sequential anthracycline-FLT3 inhibitor exposure.

## Funding Support and Author Disclosures

The authors have reported that they have no relationships relevant to the contents of this paper to disclose.
